# Supplementary material for: Oriented Insertion of ESR-Containing Hybrid Proteins in Proteoliposomes
Source: Int J Mol Sci. 2023 Apr 17;24(8):7369. doi: 10.3390/ijms24087369 (PMC10138546; doi:10.3390/ijms24087369)
Supplement: Supplementary file 1 [file ijms-24-07369-s001.zip › ijms-2317103-supplementary.pdf]

<u>Supplementary Material</u>

ORIENTED INSERTION OF ESR-CONTAINING HYBRID PROTEINS IN LIPID MEMBRANES

Lada E. Petrovskaya[a], Evgeniy P. Lukashev[b], Mahir D. Mamedov[c], Elena A. Kryukova[a], Sergei P. Balashov[d], Dmitriy A. Dolgikh[a,b], Andrei B. Rubin[b], Mikhail P. Kirpichnikov[a,b] and Sergey A. Siletsky[c1]

[a]– *Shemyakin & Ovchinnikov Institute of Bioorganic Chemistry, Russian Academy of Sciences, 117997, Moscow, Ul. Miklukho-Maklaya, 16/10, Russian Federation;*

[b]– *Department of Biology, Lomonosov Moscow State University, 119234, Moscow, Leninskie gory, 1, Russian Federation;*

[c]– *Belozersky Institute of Physical-Chemical Biology, Lomonosov Moscow State University, 119991, Moscow, Russian Federation;*

[d]– *Department of Physiology and Biophysics, University of California, Irvine, 92697, USA;*

[1]To whom correspondence should be addressed: Sergey A. Siletsky, Belozersky Institute of Physical-Chemical Biology, Lomonosov Moscow State University, 119991, Moscow, Russian Federation. Tel.: 7-495-939-5549; E-mail: siletsky@genebee.msu.su.

Table S1. Time constants and amplitudes of potential generation and changes of absorbance in the photocycle of wild type **ESR** at pH 7.5 (from [1]).

Electrogenic phases			Absorbance changes in mOD, at		
τ, ms	Amp, mV	Contribution, %	τ, ms	410 nm	590 nm
0.003	0.62	1.5	0.003	-2.1	5.6
0.024	0.70	1.7	0.0139	-0.9	1.1
0.1	0.74	1.8	0.082	-3.8	0.49
0.61	11.5	28	0.57	3.1	-0.2
3.4	19.1	46.5	2.6	2.1	-7.6
18.4	8.4	20.5	21.6	0.3	11.4

Table S2. Time constants and amplitudes of potential generation and changes of absorbance in the photocycle of **ESR-Cherry** at pH 7.5*.

Electrogenic phases			Absorbance changes in mOD, at		
τ, ms	Amp, mV	Contribution, %	τ, ms	410 nm	590 nm

0.0027	0.014	2.9	0.004	-0.55	12.3
0.079	0.04	8.4	0.09	-0.5	-1.0
0.37	-0.08	-15.4	3.4	0.8	-11.4
1.55	0.45	88.7	23.3	-0.57	14.2
7.18	-0.26	-51			

Table S3. Time constants and amplitudes of potential generation and changes of absorbance in the photocycle of **ESR-Trx** at pH 7.5*.

Electrogenic phases			Absorbance changes in mOD, at		
τ, ms	Amp, mV	Contribution, %	τ, ms	410 nm	590 nm
0.001	-0.003	-1.0	0.005	-3.4	8.2
0.028	0.02	6.7	0.128	-2.1	1.9
0.94	-0.049	-16	0.4	2.1	-3.2
1.38	0.29	93.2	4.0	1.7	-16.8
29	-0.075	-24	22.3	-0.6	26.1

Table S4. Time constants and amplitudes of potential generation and changes of absorbance in the photocycle of **Caf-ESR** at pH 7.5.

Electrogenic phases			Absorbance changes in mOD, at		
τ, ms	Amp, mV	Contribution, %	τ, ms	410 nm	590 nm
0.003	0.014	1.6	0.003	0.8	8.3
0.06	0.012	1.4	0.054	-0.5	-0.4
1.58	0.51	61	3.0	0.6	-6.0
17.6	0.31	36.4	19.7	-0.3	10.4

*Negative amplitudes in % were calculated from the sum of the positive amplitudes taken as 100%.

Table S5. Time constants and amplitudes of potential generation of the hybrid proteins at pH 6.5*.

Protein	τ1	A1	τ2	A2	τ3	A3	τ4	A4	τ5	A5	τ6	A6
wt ESR**	0.0042<br>0.032	2.1%<br>1.1%	0.58	37%	1.28	-32%	4.87	38%	14.3	22%	648	-80%
ESR-Trx	0.38	0.108	0.96	-0.171	2.49	0.146	40.55	-0.119	213	0.204	475	-0.171

		23.6%		- 37.3%		31.9%		-26%		44.5%		- 37.3%
Caf-ESR	0.0031	0.0030 1.16%	0.29	0.048 18.6%	-	-	4.2	0.122 47.3%	16.59	0.085 33%	400	-0.3 -116%

**From [2] with modifications.

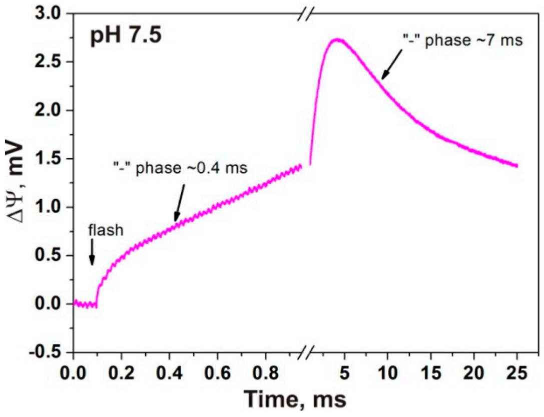

Figure S1. Kinetics of light-induced changes in membrane potential difference, ΔΨ, in proteoliposomes containing ESR-Cherry at pH 7.5.

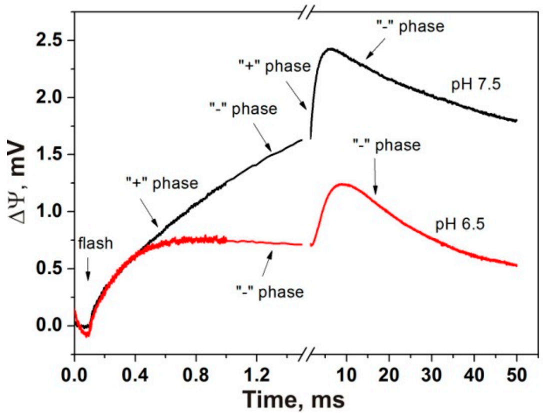

Figure S2. Kinetics of light-induced changes in membrane potential difference, ΔΨ, in proteoliposomes containing ESR-Trx at pH 7.5 and 6.5.

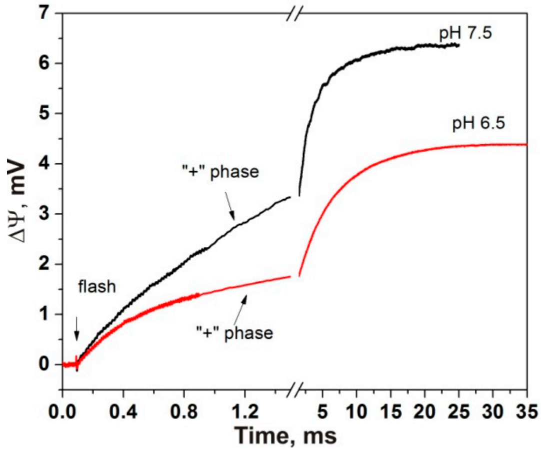

Figure S3. Kinetics of light-induced changes in membrane potential difference, ΔΨ, in proteoliposomes containing Caf-ESR at pH 7.5 and 6.5.

References

[1] S.A. Siletsky, M.D. Mamedov, E.P. Lukashev, S.P. Balashov, D.A. Dolgikh, A.B. Rubin, M.P. Kirpichnikov, L.E. Petrovskaya, Elimination of proton donor strongly affects directionality and efficiency of proton transport in ESR, a light-driven proton pump from Exiguobacterium sibiricum, Biochim. Biophys. Acta (BBA)-Bioenergetics, 1860 (2019) 1-11.

[2] S.A. Siletsky, M.D. Mamedov, E.P. Lukashev, S.P. Balashov, D.A. Dolgikh, A.B. Rubin, M.P. Kirpichnikov, L.E. Petrovskaya, Electrogenic steps of light-driven proton transport in ESR, a retinal protein from *Exiguobacterium sibiricum*, Biochim. Biophys. Acta (BBA)-Bioenergetics, 1857 (2016) 1741-1750.
